# Reusable Colorimetric Biosensors on Sustainable Silk-Based Platforms

**DOI:** 10.1021/acsabm.3c00872

**Published:** 2024-01-25

**Authors:** Augusto Márquez, Sara Santiago, Molíria Vieira dos Santos, Salvador D. Aznar-Cervantes, Carlos Domínguez, Fiorenzo G. Omenetto, Gonzalo Guirado, Xavier Muñoz-Berbel

**Affiliations:** † Instituto de Microelectrónica de Barcelona (IMB-CNM, CSIC), Bellaterra, Barcelona 08193, Spain; ‡ Departament de Química, Universitat Autònoma de Barcelona, Bellaterra, Barcelona 08193, Spain; § São Carlos Institute of Physics, University of São Paulo, São Carlos, Sao Paulo 01049-010, Brazil; ∥ Departamento de Biotecnología, Genómica y Mejora Vegetal, Instituto Murciano de Investigación y Desarrollo Agrario y Ambiental (IMIDA), 30150 La Alberca, Murcia, Spain; ⊥ Silklab, Tufts University, 200 Boston Avenue, Medford, Massachusetts 02155, United States; # CIBER de Bioingeniería, Biomateriales y Nanomedicina, Instituto de Salud Carlos III, 28029 Madrid, Spain

**Keywords:** silk fibroin, colorimetric biosensor, photoelectrochromic, dithienylethene mediators, photopatterning, point of care, glucose detection

## Abstract

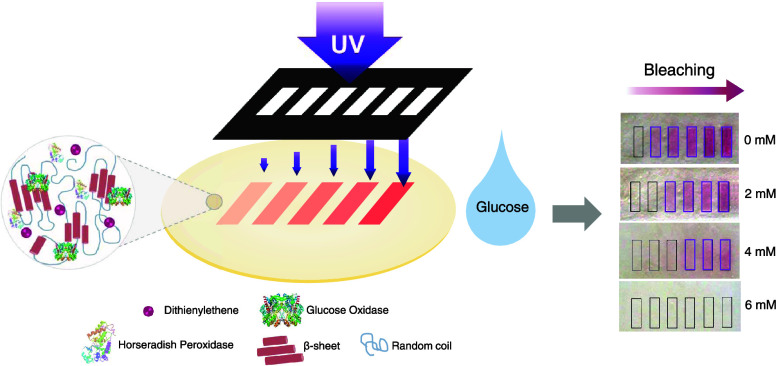

In biosensor development, silk fibroin is advantageous for providing transparent, flexible, chemically/mechanically stable, biocompatible, and sustainable substrates, where the biorecognition element remains functional for long time periods. These properties are employed here in the production of point-of-care biosensors for resource-limited regions, which are able to display glucose levels without the need for external instrumentation. These biosensors are produced by photopatterning silk films doped with the enzymes glucose oxidase and peroxidase and photoelectrochromic molecules from the dithienylethene family acting as colorimetric mediators of the enzymatic reaction. The photopatterning results from the photoisomerization of dithienylethene molecules in the silk film from its initial uncolored opened form to its pink closed one. The photoisomerization is dose-dependent, and colored patterns with increasing color intensities are obtained by increasing either the irradiation time or the light intensity. In the presence of glucose, the enzymatic cascade reaction is activated, and peroxidase selectively returns closed dithienylethene molecules to their initial uncolored state. Color disappearance in the silk film is proportional to glucose concentration and used to distinguish between hypoglycemic (below 4 mM), normoglycemic (4–6 mM), and hyperglycemic levels (above 6 mM) by visual inspection. After the measurement, the biosensor can be regenerated by irradiation with UV light, enabling up to five measurement cycles. The coupling of peroxidase activity to other oxidoreductases opens the possibility to produce long-life reusable smart biosensors for other analytes such as lactate, cholesterol, or ethanol.

## 1 Introduction

Disposable point-of-care (POC) devices have revolutionized the landscape of global healthcare by providing rapid and reliable tests able to operate out of central laboratories and by nontrained personnel. Based on the latter, a number of tests satisfying the ASSURED criteria, now REASSURED,^1^ for POC diagnostics in resource-limited regions are currently available for HIV,^2^ malaria,^3^ syphilis,^4,5^ tuberculosis,^6^ cardiovascular diseases, and type 2 diabetes mellitus.^7^ Unfortunately, access to quality diagnostics in resource-scarce areas is still limited, principally by the low stability of the biomolecules incorporated in the biosensors, that is, most POC tests are constructed using unsustainable single-use plastics, which provide poor physiological conditions for the immobilized biomolecules. As a result, these devices exhibit a low shelf life and poor accuracy when transported or stored under nonoptimal conditions.^8−10^ Best commercial devices, e.g., Onetouch, Agamatrix, or Nipro Diagnosis, ensure biosensor stability for four months in the cellulose-based matrix used as a support material, which is much more physiological than plastics. Alternatively, the use of iron oxide–mesoporous carbon nanozymes^11−13^ to substitute conventional enzymes is now being explored to increase the stability of the glucose sensors, which are of high interest in resource-limited areas.

As a step forward, we have demonstrated recently that silk films produced through silk technologies developed by Kaplan and Omenetto^14−18^ can expand the shelf life of optical enzymatic biosensors up to 8 months, even when stored dried and at room temperature.^19^ Apart from maintaining the long lifespan of biomolecules, disposable POC tests often present challenges in terms of sustainability. One of the main concerns is the significant generation of medical waste. As these tests are designed for single-use and frequently constructed from unsustainable plastics, they contribute to waste accumulation, which has the potential to harm the environment. To address this environmental issue, silk has emerged as an excellent choice for fabricating POC tests. It offers additional advantages, including biocompatibility and biodegradability, as it can degrade in soil within 8 weeks, releasing nontoxic byproducts throughout the process.^20−22^ Although they have stability and sustainability, silk-based biosensing platforms still require dedicated instrumentation for the measurement, thus compromising the REASSURED criteria for POC analysis in resource-limited settings.

In this paper, equipment-free glucose biosensors in the format of solid-state and biocompatible displays are produced by photopatterning silk films containing the enzymes glucose oxidase (GOx) and peroxidase (HRP), and photoelectrochromic molecules from the dithienylethene family as mediators. In a previous publication,^23^ we have demonstrated that 1,2-bis(5-carboxy-2-methylthien-3-yl)cyclopentene (DTE) can substitute traditional end-point enzymatic mediators in the production of resettable optical glucose biosensors that may be reused several times. The measurement principle is based on the selective reaction of the closed isoform of DTE (pink) by HRP in the presence of the substrate, either hydrogen peroxide or glucose, when coupled to GOx activity. During the reaction, the closed pink isoform is converted into its opened and uncolored isomer, which can be regenerated after the enzymatic reaction by a short exposition to low-energy UVB (λ = 312 nm) light radiation (30 s). Therefore, the properties of the photoelectrochromic mediator enable (i) the optical determination of glucose concentration by a visual color change and (ii) restoration of the initial conditions of the biosensor to perform multiple glucose measurements. The same principle is exploited here in the development of a solid-state colorimetric glucose biosensor on silk films. The production of the biosensor involves (i) the incorporation of the enzymes and mediators into the aqueous silk fibroin (SF) solution; (ii) the crystallization of the doped SF aqueous solution through water vacuum annealing to produce silk films; and (iii) the photopatterning of the silk films to produce simple solid-state enzymatic biosensors, which are readable with the bare eye. Silk biosensors are tested in buffer samples to determine their potential use in resource-limited regions. The response of the biosensor is evaluated with the bare eye and compared to reference methods. The reusability and recyclability of the platform, combined with the fact that it is made of biodegradable and nontoxic silk, make this system ideal within the framework of environmental sustainability.

## 2 Materials and Methods

### 2.1 Reagents

Phosphate buffered saline (PBS, 10×), ethanol (absolute for analysis), d-(+)-glucose (≥99.5%), sodium carbonate (Na_2_CO_3_, ≥99.5%), lithium bromide (LiBr, ≥99%) peroxidase from horseradish (HRP; Type VI-A, essentially salt-free lyophilized powder, 250–330 U mg^–1^), and glucose oxidase from *Aspergillus niger* (GOx; Type X-S, lyophilized powder, 100–250 U mg^–1^) were purchased from Sigma-Aldrich. Diacid dithienylethene 1,2-bis(5-carboxy-2-methylthien-3-yl)cyclopentene (DTE) was synthesized following a procedure previously described in the literature.^24−27^ All chemicals were used as received, and aqueous solutions were prepared using deionized water with a resistivity of 2 MΩ cm^–1^.

### 2.2 Silk Fibroin Processing

Cocoons of *Bombyx mori* were chopped into four or five pieces and boiled in 0.02 M Na_2_CO_3_ for 30 min to remove the glue-like sericin proteins. Then, raw fibroin was rinsed thoroughly with water and dried at room temperature for 3 days. The extracted fibroin was dissolved in 9.3 M LiBr for 3 h at 60 °C to generate a 20% w/v solution that was dialyzed against distilled water for 3 days (Snakeskin Dialysis Tubing 3.5 kDa MWCO, Thermo Scientific) with eight total water exchanges, resulting in a 7–8% w/v fibroin solution. The salt-free solution was concentrated up to 20% w/v by water evaporation, maintaining the solution in the dialysis membranes in a dry atmosphere.

### 2.3 Doping of SF Films with the Enzymes and DTE Mediator

The production of the silk biosensors^22,23^ required the incorporation of the enzymes GOx and HRP and the mediator DTE in the silk matrix. Water-soluble GOx (25 μg mL^–1^) and HRP (60 μg mL^–1^) were directly dissolved in the 20% w/v SF aqueous solution as already reported.^16^ Due to its high insolubility in water, DTE could not be dissolved directly but required a presolubilization step in ethanol. DTE was thus dissolved in ethanol up to a concentration of 1.2 × 10^–3^ M, corresponding to its maximum solubility in this medium. This solution was mixed with an equivalent volume of H_2_O and then added to an equivalent volume of 20% w/v SF. Water-soluble SF in contact with alcoholic solutions tended to aggregate and precipitate in nontransparent SF hydrogels.^24^ For this reason, an intermediate dilution of DTE–ethanol solution with water was crucial to reduce the SF gelation velocity. The dilution in water slowed the formation of the hydrogel, maintaining the stability of the SF mixture for 2 h. To obtain transparent SF films, the final SF precursor solution containing 3 × 10^–4^ M DTE in 10% w/v SF (ethanol–H_2_O relation: 1:3) should be processed within the first 2 h after preparation. In the processing, the precursor solution was cast onto polystyrene flat surfaces and evaporated at 60 °C for 30 min to eliminate any trace of ethanol.

Notice that in this study, ethanol is used for the presolubilization of the DTE compound and not for the crystallization of fibroin. In this scenario, ethanol does not induce the formation of β-sheet structures since it comes into contact with the fibroin when it is dissolved in water. Note that the induction of β-sheet structures through the use of organic solvents occurs only when, after evaporating the water from the fibroin solution (forming films), it comes into contact with ethanol.

### 2.4 Silk Film Biosensor Production and Testing

Silk biosensors were produced by selective photopatterning of the previous enzymatic silk films. Photomasks for the patterning were produced in-house either (i) by printing the patterns with a laser printer (Xerox B210) in 100 μm-thick polyester transparent A4 sheets from APLI S.L. (Alaquàs, Spain) or (ii) through laser ablation with a CO_2_ laser writer (Epilog Mini 24, Epilog Laser) of opaque pressure-sensitive adhesive (PSA) films (ARcare 8939, Adhesives Research Inc., Glen Rock, PA). The photolithographic process involved the alignment of the photomask on top of the silk film and its subsequent irradiation with UV light (i.e., λ_exc_ = 312 nm; 12 W power). UV light irradiation produced the photoisomerization of the DTE molecules present in the exposed regions and their color change from uncolored to pink. Since only the exposed regions change color, it was possible to produce color patterns by selective exposition with the photomask (i.e., pattern transference). Additionally, the photoisomerization process was dose-dependent and longer exposition times and/or higher light intensities resulted in stronger color intensities. This property was employed to produce patterns with different color intensities that enabled glucose detection without the need for external instruments but through simple visual inspection. Two biosensor configurations were produced for glucose sensing, which differed on the photopatterning strategy. In the first case, printed photomasks were used, consisting of a single rectangular region printed with a color gradient (i.e., gray scale, from transparent to black). This color gradient produced an increasing opacity of the pattern, reducing the amount of light reaching the silk film and thus the irradiation dose. This resulted in a rectangular pattern with decreasing pink color intensity proportional to the level of photoisomerization, obtained through a single photolithographic step.

In the second case, six rectangular apertures of 3 mm × 5 mm separated by 1 mm were produced by laser ablation on the photomask. Each rectangular aperture was irradiated at different times (i.e., 0, 10, 20, 30, 40, and 50 s). As a result of the irradiation process, a pattern with rectangular colored areas of increasing intensities was obtained. The 50 s irradiation coincided with the photostationary state of the compound, which corresponded to the maximum color intensity available for the system.

After irradiation, the sensing capacity of the activated material was evaluated with glucose solutions prepared 24 h in advance to ensure the equilibrium shifts toward the β-d-glucose form. Silk films were brought into contact with absorbent filter paper soaked in various glucose concentrations (0, 2, 4, and 6 mM). The filter paper was cut to the same dimensions as the patterned silk membrane and saturated with different glucose solutions until the paper was completely moistened and a slight leakage began to be observed. The total volume of the solution used at that point was 800 μL. The following step involves taking photographs at different time intervals (up to 14 min) and analyzing the images using ImageJ.

### 2.5 Spectral Imaging

For the spectral image analysis, an Olympus inverted microscope, equipped with a halogen lamp (Olympus, U-LH100L-3) as a light source and coupled to a multispectral camera (CRI Nuance EX) controlled using Nuance 3.0.2 software, was used in the characterization of the silk films doped with DTE. Images were acquired and unmixed in the different spectral components. The absorbance magnitude was obtained by comparison between a reference area (non-UV-irradiated silk) and the area under study (UV-irradiated silk).

In the analysis of the silk biosensors, images were acquired using a Canon EOS 250D in a photobox illuminated with a cool white LED (6000 K; 18 W power) to avoid the interference of the environmental light. Images were then analyzed using ImageJ. First, they were split into the three primary color channels (i.e., red, green, and blue), only considering the green one for the analysis since it presents the highest sensitivity to the measurement. The rectangle tool was then used for the selection of the colored areas to be analyzed, ignoring the edges.

## 3 Results and Discussion

### 3.1 Production of Photochromic Enzymatic Silk Films

**Figure 1:**
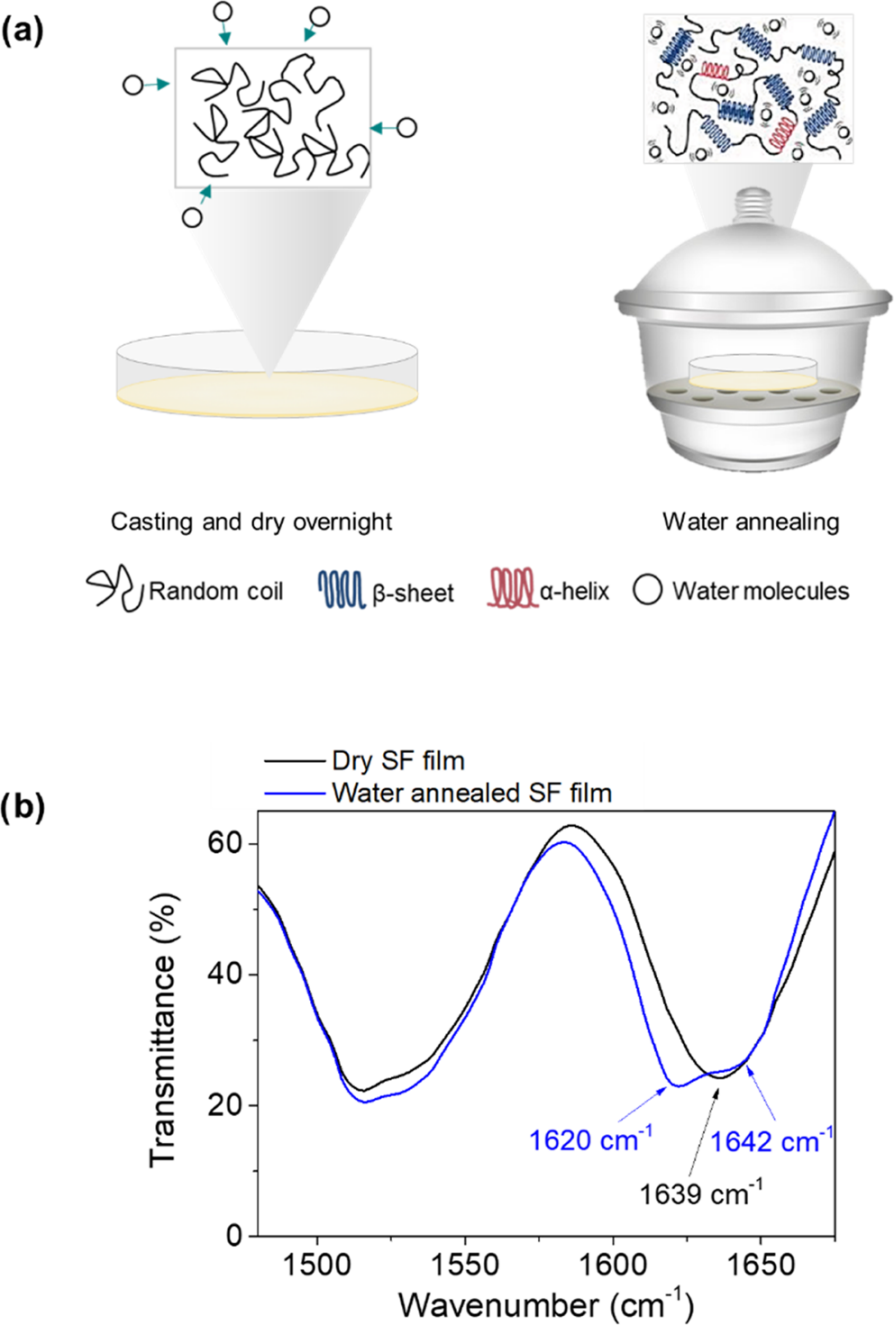
Formation of crystalline enzymatic silk films doped with DTE. (a) Illustration of the crystallization protocol, involving casting, drying, and water annealing in the vacuum. (b) ATR-FTIR spectra of dried (black) and crystalline silk films (blue).

Enzymatic silk films were prepared with the previous protocol by drying and crystallizing the precursor silk solution resulting from mixing the SF aqueous solution containing the enzymes with the water–ethanol solution of the DTE mediator (Figure 1a; more details are in the Supporting Information (SI), Figure S1). The crystallization process of the resulting material was evaluated by attenuated total reflectance Fourier-transform infrared (ATR-FTIR) spectroscopy (Figure 1b). The presence of a peak at 1620 cm^–1^ in the ATR-FTIR spectra confirmed the formation of β-sheets and the correct crystallization of the doped silk films.^19^ The crystalline structure of the films was very important to ensure their insolubility and the stability of the enzymes and mediators in their nanoporous matrix. The enzymes in the solution maintained 90% activity after the film formation, which expanded for more than 8 months, as demonstrated in ref (19).

**Figure 2:**
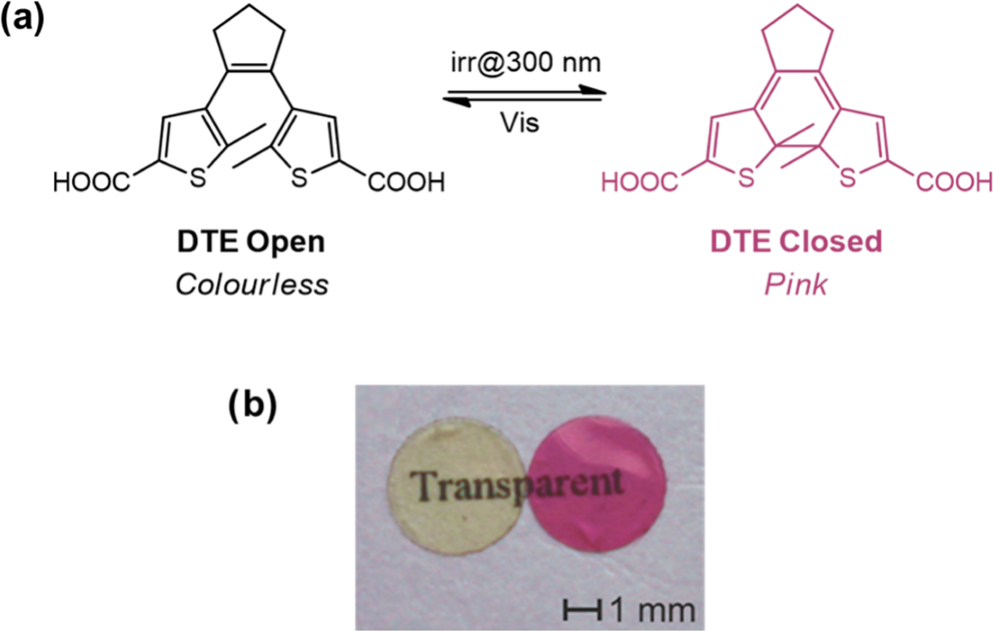
Enzymatic silk films with photochromic activity. (a) Isomerization reaction of DTE molecules. The ring opening/closing of the structure is accompanied by a color change from transparent to pink when irradiated with UV. The reaction is reversible using visible light. (b) Immobilized DTE in enzymatic silk films containing GOx and HRP keeps the photochromism. The pads were placed over the printed word “Transparent” to denote the transparency of the film.

On the other hand, the presence of a DTE mediator conferred the enzymatic silk films with photochromic properties and sensitivity to glucose. As shown in Figure 2a, DTE presented two photoisomeric forms: the open, which was uncolored, and the closed one, with an evident pink coloration.

After film formation, circular fragments of 4 mm were cut by laser ablation. Initially, the only existing isomer in the film was the open one, which was photoisomerized after 30 s of irradiation with UV light at 312 nm (Figure 2b). The high concentration of DTE in the film conferred an intense pink coloration. Then, the opposite reaction could be reversibly induced by irradiation with visible light or simply with ambient light without the need for irradiation.

Additionally, the color of the film was uniform, suggesting a homogenous distribution of the DTE on the SF matrix. The color change also demonstrated that DTE molecules could be photoisomerized in the SF matrix as in the solution, without suffering from steric hindrance and allowing the conformational rotation of the isomerization reaction.

### 3.2 Enzymatic Silk Film with Sensitivity to Glucose

**Figure 3:**
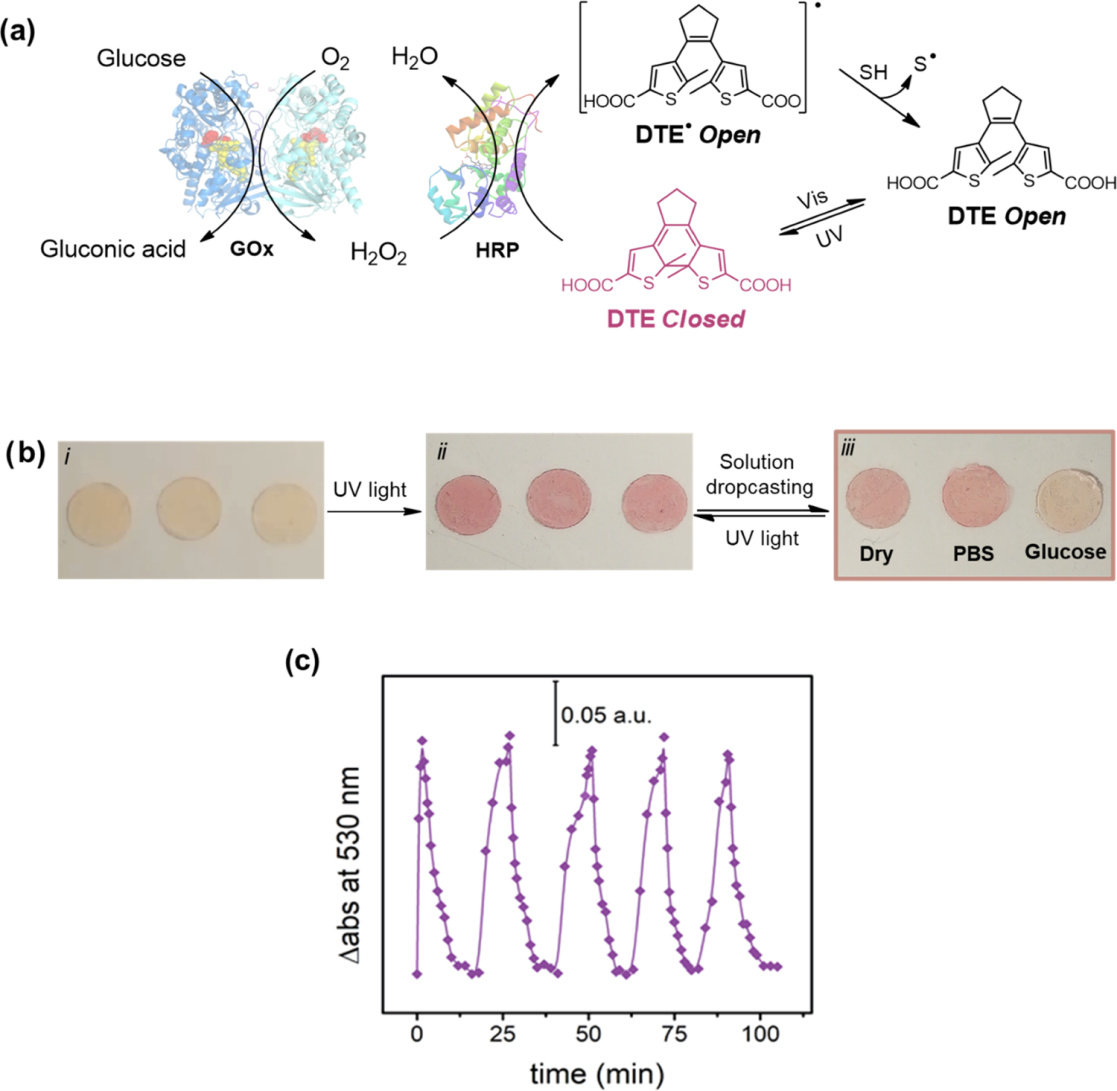
DTE cycle in enzymatic silk films. (a) Enzymatic cascade reaction where DTE acts as a reversible colorimetric mediator activated via UV light in the enzymatic silk films. SH represents a protic solvent, and S^•^ represents the corresponding radical species. (b) Nonirradiated doped SF pads (i) were exposed to UV for 30 s to close the DTE molecule resulting in the change of color (ii). One of the pads was maintained dry as a control, while a drop of PBS solution and a drop of 4 mM glucose PBS solution were added on top of the others (iii). After 10 min, only the pad in contact with the glucose solution lost the color. (c) Color of the enzymatic silk films was restored after a second UV irradiation in all samples. This process could be repeated a minimum of five times.

In a previous publication,^23^ we demonstrated that the isomerization of DTE from the closed to the open form could be induced enzymatically in solution (Figure 3a). Briefly, GOx from *Aspergillus niger* is well known to specifically catalyze the reaction between glucose and oxygen to produce d-glucono-1,5-lactone and H_2_O_2_. This reaction is very specific since it is based on the selective activity of an enzyme, which was used as biocatalysts in the biosensor. The H_2_O_2_ was then selectively reduced to O_2_ and H_2_O by another enzyme, i.e., HRP, which, at the same time, oxidized the close DTE to a radical form by the loss of a hydrogen atom, following a 1:1 stoichiometry. This form finally evolved to the more stable radical open form that may eventually be hydrogenated with radical species from the protic solvent, resulting in the formation of the open form.

The interaction between DTE and HRP was isomer-selective, and only the closed DTE form could be oxidized to the radical intermediate in the presence of H_2_O_2_ or glucose, the latter after coupling the previous reaction with GOx activity. This isomer selectivity and the formation of the radical intermediate form were demonstrated in ref (23) by voltammetry, confirming that (i) the open and closed forms of the DTE showed different reduction potentials and only the closed one presented a potential low enough to be oxidized by HRP and (ii) the reaction between HRP and the closed DTE generated a new transparent species with a different voltammetric peak from the open or closed DTE forms. As a result, the enzymatic silk film doped with DTE should lose its initial pink coloration in the presence of glucose. However, to demonstrate that the discoloration results from the enzymatic activity and not from photo- or thermal back-isomerization processes, a proof-of-concept assay was conducted with three circular enzymatic silk films, which were incubated at different experimental conditions (Figure 3b).

All samples were first irradiated at 312 nm for 30 s, resulting in similar color and thus DTE close form concentration. After that, one sample was kept dried (Figure 3b, left), the second was incubated with PBS and used as a negative control of the enzymatic reaction (Figure 3b, middle), and the third was incubated in a PBS solution containing 4 mM glucose (Figure 3b, right). Results after 10 min of incubation (Figure 3b(iii)) showed that only the sample in contact with glucose lost color over time as a consequence of the cascade enzymatic reaction detailed before.

Additionally, the photoisomerization kinetics was evaluated, observing important difference between the ring closing (from transparent to pink) and the ring opening (from pink to transparent), as already reported in previous publications for DTE molecules in solution.^28^ The closing process induced by UV light irradiation was very fast and only required 2 min to reach the maximum color intensity (i.e., total DTE conversion). On the contrary, the opening process was much slower, and films needed around 24 h of exposure to ambient light to bleach. The slow decoloration kinetics upon visible light of the enzymatic silk films increased their stability and applicability, for example, in the production of reusable glucose biosensors. In this sense, bleached films were reused for a second analysis after a short irradiation with UV light (30 s), which induced photoisomerization, restoring the initial color of the SF film and its biosensing capacity (Figure 3b(ii)) in a process that could be repeated a minimum of five times (Figure 3c). This result agreed with the previous hypothesis that the radical open DTE form spontaneously reacted with the solvent, recovering the initial open DTE form, which was susceptible of photoisomerization to the closed DTE by irradiation. In terms of sensor performance, DTE molecules kept the reversible photoisomerization capability previously observed in solution and presented similar opening/closing kinetics both in solution and in the SF matrix. This observation indicated that the DTE mediator did not suffer from steric hindrance once immobilized in the SF biopolymeric matrix, where it remained stable for multiple glucose detection cycles. It should be noted, however, that the sensor performance was strongly influenced by the DTE ring opening–closing process in terms of detection time and sensor stability. On the one hand, 10 min was necessary to reach detectable levels of the open DTE form visually, while it only required 30–50 s of irradiation to close the DTE ring and restore the initial conditions of the sensor. This opening/closing kinetics was slower than simple electron transference steps occurring with inorganic redox mediators, increasing the sensor response time. On the other hand, the mediator photoisomerization enabled restoration of the biosensor and used it multiple times, but for applications requiring long storage or measurement periods, silk films doped with DTE should be kept in the dark to minimize spontaneous opening/closing processes affecting the sensor performance.

### 3.3 Production of Silk-Based Biosensors through Photopatterning

**Figure 4:**
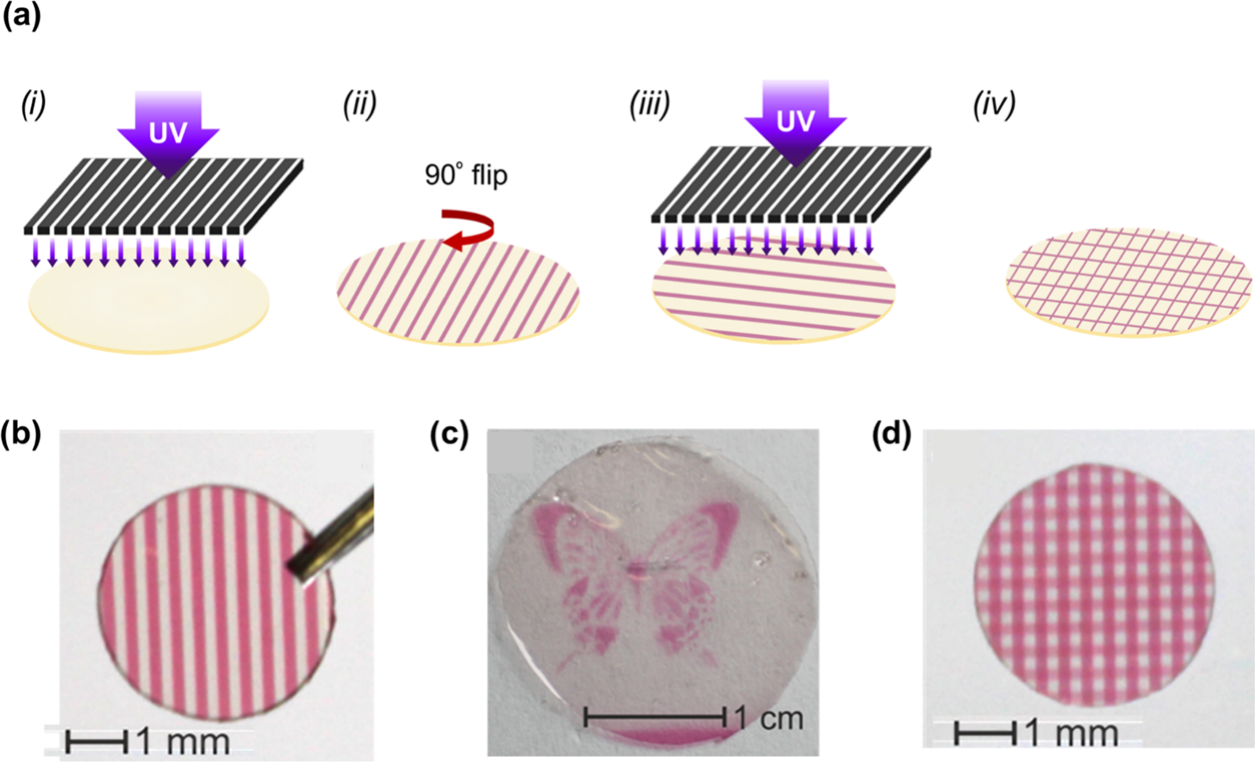
Photolithographic patterning of enzymatic silk films doped with DTE. (a) Photopatterning steps on SF functionalized with DTE, GOx, and HRP. After the first UV pattering, the same pattern is developed again on the film after turning the mask 90°. (b) Photographs of 4 mm-diameter SF films doped with DTE and enzymes after 30 s of UV irradiation using a photomask with straight lines. (c) It is possible to develop figures or regular patterns on the films using a suitable mask during the UV irradiation. (d) Photograph of 4 mm-diameter SF films after two consecutive irradiation steps with the same photomask, after shifting the photomask 90° in the second irradiation step.

The specific photoisomerization of the DTE molecules enabled the patterning of the enzymatic silk films. Circular portions of doped silk films were cut and irradiated with UV light at 312 nm (30 s) through a photomask containing specific patterns (Figure 4a(i)). The patterns in the photomasks were transferred to the silk film with high precision and spatial resolution (below 200 μm, which corresponded to the thickness and separation of the lines in the mask) through a simple photolithographic step, either regular and repetitive arrangements (Figure 4b) or complex and irregular patterns such as a butterfly (Figure 4c). The photoisomerization process was, moreover, dose-dependent; the degree of photoisomerization of the molecule and thus the color intensity of the pattern could be modulated by adjusting the irradiation dose, e.g., the irradiation intensity or irradiation time. Based on this principle, a coloration gradient was created after two successive UV irradiation steps of the same regular pattern by shifting the photomask 90° (Figure 4a(i–iv)). As shown in Figure 4d, the regions exposed twice presented a more intense pink color (double color intensity) than those exposed only once.

**Figure 5:**
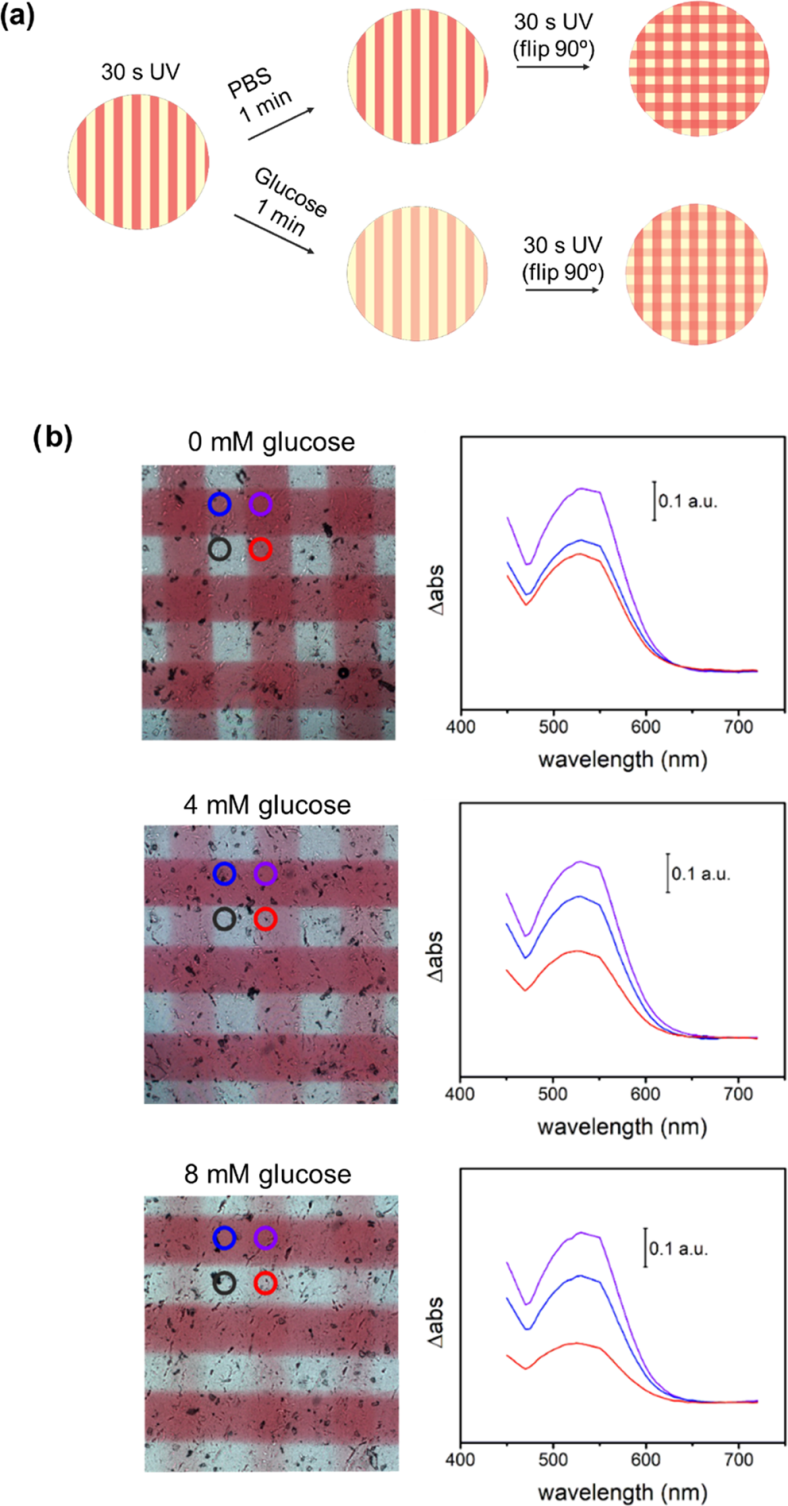
Glucose detection using patterned enzymatic silk films. (a) First, a doped SF pad of 4 mm diameter is irradiated with UV for 30 s, using an aluminum mask to generate patterns of 200 μm. Then, the pad is incubated in PBS aqueous solution for 1 min with 0, 4, and 8 mM glucose. Finally, the pad is removed from the solution, dried, and irradiated using the same mask forming 90° angle with respect to the previous pattern. (b) On the left, microscopy images of silk pads in contact with different glucose solutions in PBS (0, 4, and 8 mM from top to bottom). The glucose PBS solution led to different color decays within the film. By image spectral analysis (on the right), it is possible to quantify the decay in different areas of the film: never irradiated area (black), irradiated area and incubated in 0, 4, or 8 mM glucose solution (red), irradiated area after solution removal (blue), and irradiated area before and after the immersion (purple).

To evaluate the potential of photopatterned silk films in glucose biosensing, an initial experiment was performed as a proof of concept, which is illustrated in Figure 5a. As before, 4 mm circular pads were cut from the enzymatic silk films and exposed to UV light radiation for 30 s through a photomask with a periodic pattern of 200 μm. The photopatterned silk films were then immersed in PBS solutions with glucose (0, 4, or 8 mM) for 1 min, and after the removal of the liquid excess, they were exposed again for 30 s to UV irradiation after rotating the mask 90° with respect to the previous one.

The resulting photopatterned silk films are presented in Figure 5b. In the case of PBS solutions without glucose (Figure 5b, top), the areas irradiated once (either during the first or second photolithographic step) presented similar absorbance magnitude at 530 nm corresponding to the maximum absorbance of the closed isoform of DTE. Conversely, those areas common in both photolithographic steps were irradiated twice and presented absorbance values that doubled the previous ones. This demonstrated (i) the dose-dependent photoisomerization of DTE, even during sequential irradiation steps, and (ii) the null or negligible leakage of DTE molecules from the SF matrix to the medium when immersed in aqueous solutions.

The results differed when glucose was added to the PBS solution during the incubation step. Two glucose concentrations were studied, i.e., 4 mM (Figure 5b, middle) and 8 mM (Figure 5b, bottom), which corresponded to normoglycemic and hyperglycemic levels, respectively. As before, after incubation, a second photolithographic stage was performed where the photomask was rotated 90° versus the first one. The presence of glucose during the incubation step resulted in a clear decrease in the color intensity of the first pattern, which was associated with the enzymatic oxidation of the closed form of DTE when the enzymatic cascade was activated. This color loss was quantified spectroscopically, taking the nonirradiated regions as a reference spectrum. Moreover, the color loss, either by visual inspection or spectroscopic analysis, was proportional to the glucose concentration in the PBS solutions, enabling glucose quantification in an equipment-free manner. Based on this principle, two silk film glucose biosensors were produced and tested, namely, gradient biosensors and multipoint biosensor displays.

### 3.4 Gradient Biosensors

**Figure 6:**
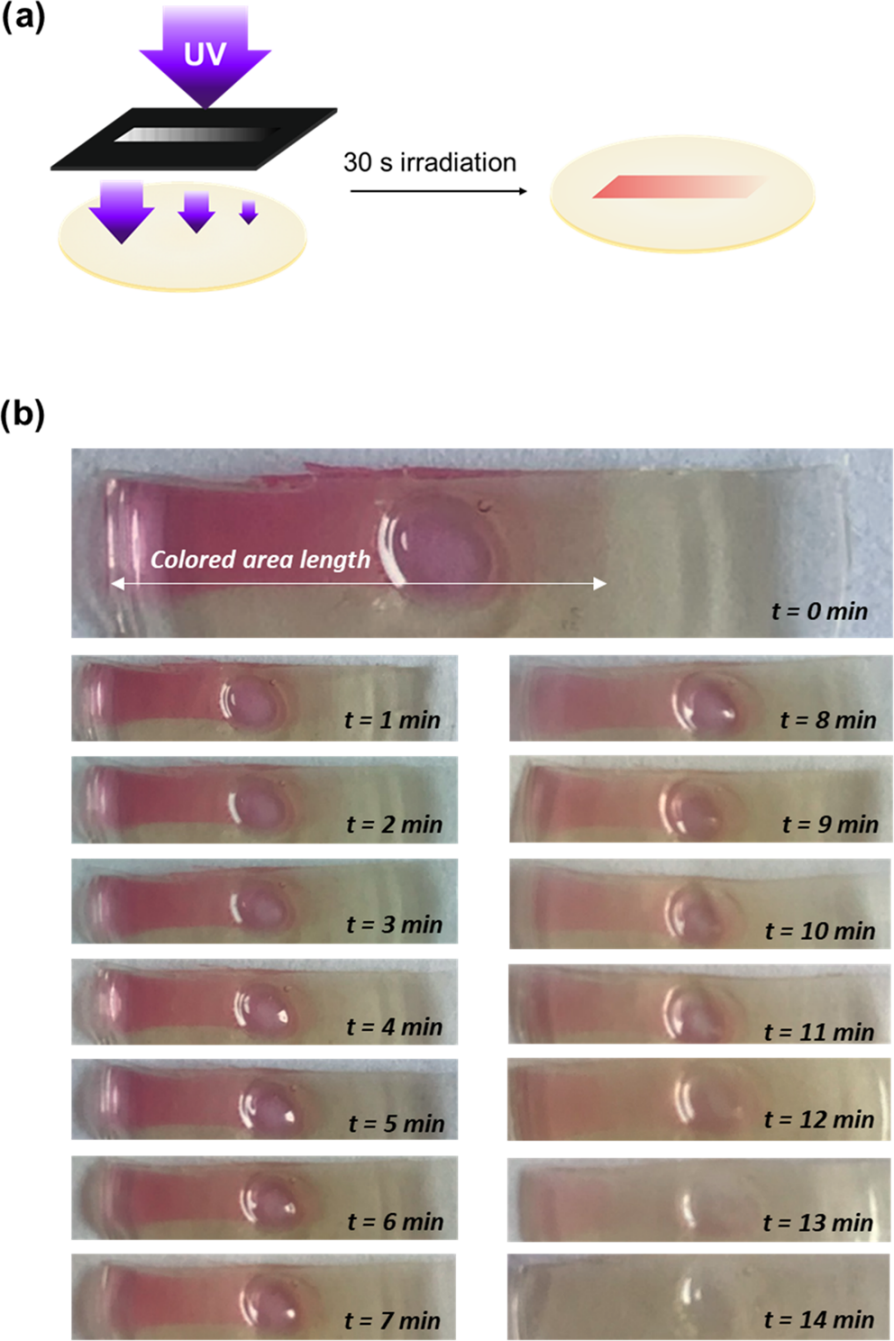
Gradient biosensor production and performance. (a) Patterning was done using a mask with a gradient of opacity to UV light. For 30 s, the SF enzymatic film was irradiated and a gradient of color was revealed on the surface. (b) Images corresponding to a sample photopatterned following procedure (a) and placed in contact with a 4 mM glucose solution in PBS for 14 min. It can be noted that the bleaching color takes places gradually from the less-intense colored side to the most intense along time.

Gradient biosensors were produced by irradiation of photoelectroenzymatic silk films through a printed photomask consisting of a rectangle with a gradual decreasing opacity. The biosensor production is illustrated in Figure 6a, where a pink region with a color gradient resulting from the dose-dependent DTE photoisomerization is clearly observed. The fabrication involved a single photolithographic step, a process requiring less than one min that ensured a simple, fast, and scalable manufacturing of the biosensor. The analytical capacity of the biosensor was evaluated by incubation with 4 mM glucose samples. During incubation, a progressive decoloration of the silk films was observed over time, which was associated with the activation of the enzymatic reaction, resulting in the oxidation and bleaching of the DTE molecules (Figure 6b). From a kinetic point of view, the regions with stronger color intensities required longer incubation times, 14 min being necessary for the total decoloration of the biosensor. Although the decoloration kinetics depended on the initial glucose concentration, it was not possible to determine the glucose concentration with this configuration since the transition point between colored and uncolored areas was blurry and difficult to assess with precision.

### 3.5 Multipoint Biosensor Displays

**Figure 7:**
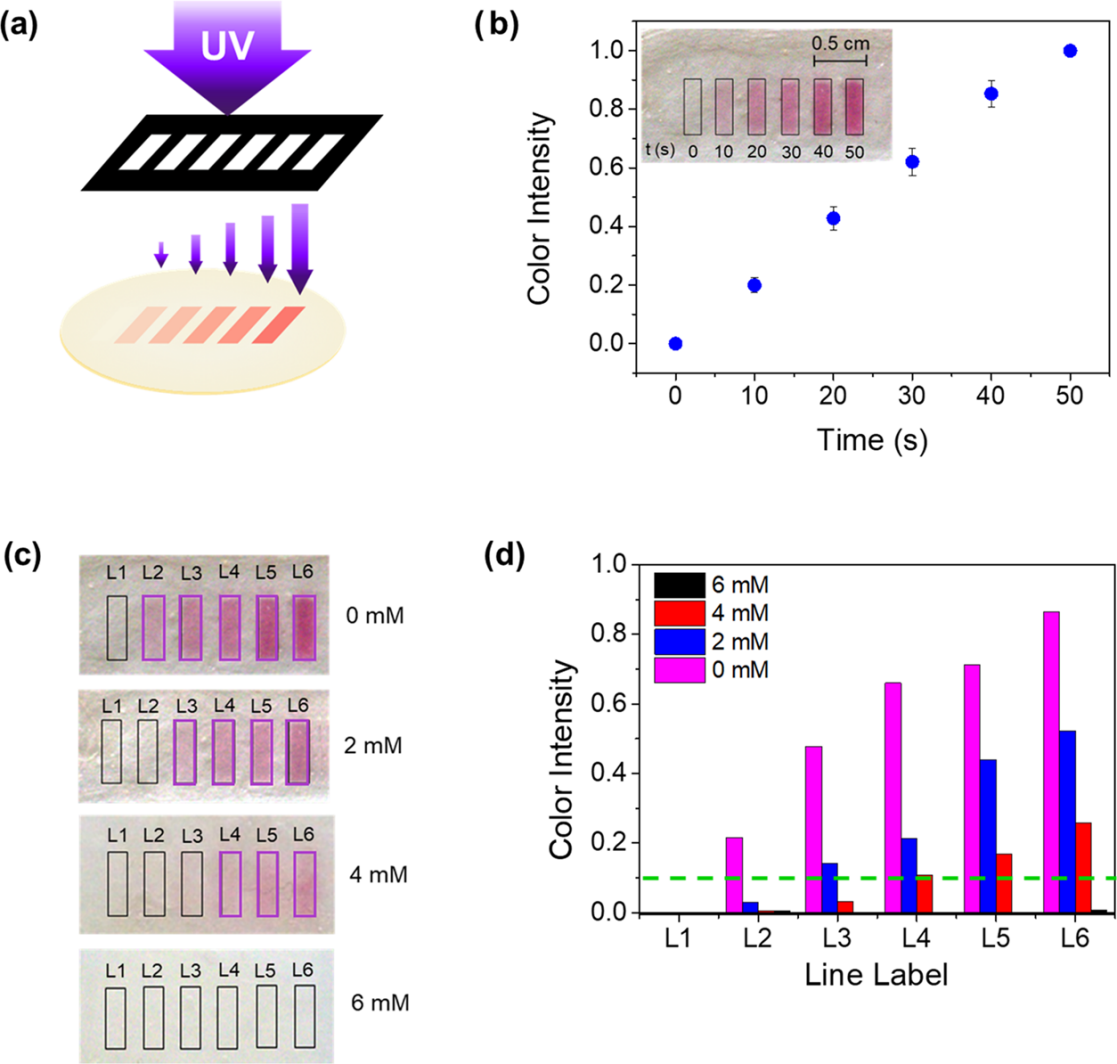
Quantification of glucose levels using a photopatterned silk film display. (a) Scheme with the photolithographic procedure for the production of colorimetric silk displays enabling glucose levels quantification. Each rectangle is irradiated with increasing doses of UV light (from left to right). (b) Color of each rectangle after the irradiation doses was analyzed using ImageJ software. After splitting the image by colors, the green channel was selected for being the one providing the best sensibilities. (c) Eventually, four films were patterned identically and used to sense different concentrations of glucose in PBS (0, 2, 4, and 6 mM). The picture shows the color variation after 30 min on the displays. In pink, the last rectangle observable with the naked eye is shown. (d) Graph shows the bleaching color of each rectangle after the incubation in glucose solutions. Images were analyzed with ImageJ following the previous procedure. The green dashed line indicates the color intensity (0.1 au) at which it is possible to detect the bleaching color on the display.

In an attempt to enhance color contrast in the transition area, multipoint biosensor displays were produced. These biosensor displays consisted of six rectangles with increasing color intensities separated by an uncolored region. The rectangular geometry of the colored regions simplified the identification of the transition areas, being simpler to decide if one region was still colored or not. For the production of the biosensor (Figure 7a), each consecutive region was exposed during longer irradiation times, which corresponded to larger irradiation doses. As shown in Figure 7b, color intensities were directly proportional to the irradiation times, which confirmed the linear relationship between irradiation doses and DTE photoisomerization ratio.

Four glucose concentrations in PBS were studied, corresponding to the control (0 mM), hypoglycemic (2 mM), normoglycemic (4 mM), and hyperglycemic (6 mM) glucose levels (Figure 7c,d). Color bleaching associated with the enzymatic oxidation of DTE by HRP only occurred after incubation with samples containing glucose, while the control sample without glucose remained stable over time. As in the previous gradient biosensor, DTE discoloration was progressive over time and dependent on the glucose concentration (Figure 7d), that is, regions with weaker color intensities disappeared first. On decoloration, the kinetics was proportional to the initial glucose concentration in the sample.

As a result, the number of regions decolored over time depended on the initial glucose concentration on the sample, which may be used to quantify the glucose level without the need for external instruments as illustrated in Figure 7c. Just counting the number of colored lines still remaining in the biosensor display after 30 min of incubation, it is possible to distinguish between a hypoglycemic (four lines), a normoglycemic (three lines), or a hyperglycemic (zero lines) situation. When compared to the results obtained in Section 3.4, longer incubation times were necessary in this case due to the extended exposure of the material to UV light, resulting in a higher concentration of the catalytic form of DTE (the closed and colored form).

Thus, the multipoint biosensor display configuration allowed the determination of glucose levels by simple visual inspection. The process was very simple and only required counting the number of colored lines in the display after 30 min of incubation (response time = 30 min) for sample concentrations between 2 mM and 6 mM (limit of detection = 2 mM). The resolution of the biosensor, understood as the minimum concentration difference detectable, was 2 mM. It is important to remark that all these parameters may be adjusted and improved by modifying the biosensor architecture. For example, modifying the irradiation time to smaller doses should reduce the decoloration time of the lines and thus the time to obtain the result. Also, adjusting the enzyme/mediator concentration may be possible to increase the sensitivity and/or the detection range of the sensor.

In summary, the combination of SF matrix supports, biocatalysts, and DTE as a redox mediator resulted in novel biosensor configurations exhibiting high specificity (by the use of enzymes), large stability comparable to inorganic nanozymes (due to the enhanced stability of enzymes when incorporated in the SF matrix), and reusability (understood as the capacity to perform multiple measurements) thanks to the photoisomerization activity of the DTE molecules. Other platforms like those based on bacterial cellulose^11^ or mesoporous flexible materials^29^ may present similar advantages in terms of biocompatibility, sustainability, and entrapment capacity. However, the capacity of SF to stabilize enzymes and maintain them active for long time periods is the longest reported up to now and may be the principal advantage of the current biosensing platform.

## 4 Conclusions

The homogenous immobilization of the poorly water-soluble photoelectrochromic DTE within the SF was achieved by the combination of water–ethanol mixtures of the DTE and SF solutions before deposition and a fast solvent evaporation to obtain transparent films rather than gels. The capability of the molecule to undergo reversible isomerization in the solid-state SF films was demonstrated by UV irradiation, with no evidence of particle aggregation according to the transparency and homogenous color change of the films. The coimmobilization of the enzymes GOx and HRP with the DTE inside the SF films conferred them with biosensing capacity and specificity to the analyte of interest. Closed (colored) DTE, which was previously demonstrated to be sensitive to HRP catalysis in the presence of H_2_O_2_, lost its color when the doped SF films were immersed in buffer solutions containing glucose. This fact was used for the development of optical biosensors through fully sustainable and water-based technologies, which are able to display glucose levels without the need for external instrumentation and to satisfy the REASSURED criteria for the development of point-of-care systems for resource-limited regions. Two configurations were produced, i.e., gradient biosensors and multipoint biosensor displays, which were manufactured by selective photopatterning protocols taking advantage of the dose-dependent photoisomerization of DTE molecules in the SF films. Thus, increasing UV dosages resulted in larger photoisomerization ratios in the silk films, which required longer incubation times with glucose samples to return to the uncolored open form of DTE. This concentration-dependent discoloration process enabled us to distinguish between hypo-, normo-, and hyperglycemic levels through simple visual inspection. Besides, the sensors could be regenerated after UV irradiation, and it was possible to reuse them at least five times. Since the H_2_O_2_ production can be coupled to other oxidoreductases, it may be possible to produce biosensor displays based on the same measurement principle but selective to other analytes such as lactate, ethanol, cholesterol, or succinate, among others.

## Supporting Information Available

Details regarding the silk fibroin doping process with DTE (PDF)
