# Supplementary material for: Reusable Colorimetric Biosensors on Sustainable Silk-Based Platforms
Source: ACS Appl Bio Mater. 2024 Jan 25;7(2):853–62. doi: 10.1021/acsabm.3c00872 (PMC10880051; doi:10.1021/acsabm.3c00872)
Supplement: Supplementary file 1 — mt3c00872_si_001.pdf [file mt3c00872_si_001.pdf]

Supporting Information

Reusable Colorimetric Biosensors on Sustainable Silk-Based Platforms

Augusto Márquez[a,$], Sara Santiago[a,b,$], Molíria Vieira dos Santos[c], Salvador Aznar[d], Carlos Domínguez[a], Fiorenzo G. Omenetto[e], Gonzalo Guirado[b,*], Xavier Muñoz-Berbel[a,f,*]

[a]Instituto de Microelectrónica de Barcelona (IMB-CNM, CSIC), Bellaterra (Barcelona), 08193, Spain

[b]Departament de Química, Universitat Autònoma de Barcelona Bellaterra (Barcelona), 08193, Spain

[c]São Carlos Institute of Physics, University of São Paulo, São Carlos, SP, Brazil

[d]Departamento de Biotecnología, Genómica y Mejora Vegetal, Instituto Murciano de Investigación y Desarrollo Agrario y Ambiental (IMIDA), 30150, La Alberca, Murcia, Spain

[e]Silklab, Tufts University, 200 Boston Avenue, Medford, MA 02155, USA

[f]CIBER de Bioingeniería, Biomateriales y Nanomedicina, Instituto de Salud Carlos III.

[$]Both authors contribute equitably

*Corresponding Authors: email: xavier.munoz@imb-cnm.csic.es; email: gonzalo.guirado@uab.cat

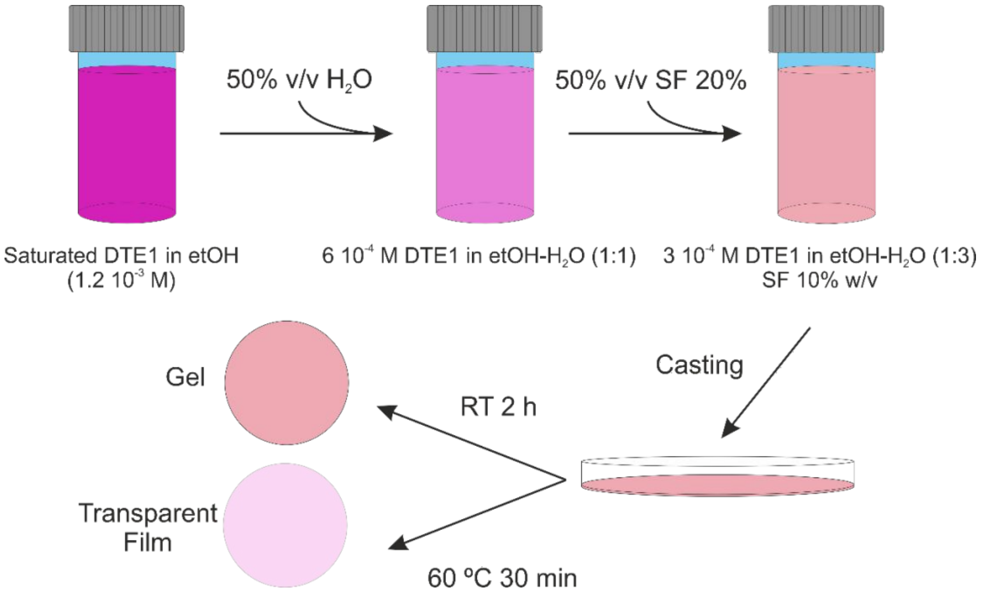

Figure S1. Silk Fibroin doping process with DTE. The supernatant of a saturated solution of closed DTE (1.2 10^{-3} M) in ethanol was mixed with the equivalent volume of H_2O. The 1 to 1 etOH-H_2O solution of DTE1 was then mixed with an equivalent volume of 20 % w/v of silk fibroin aqueous solution. A fast evaporation of the solvent drove to the formation of a transparent film while the incubation at room temperature carried to a gel formation.
